# Body Part Pain Affects Subjective and Objective Handball Performance in Japanese Male National Athletes—Results of Short-Term Practical Monitoring of Athletes’ Conditions

**DOI:** 10.3390/sports12030065

**Published:** 2024-02-21

**Authors:** Issei Ogasawara, Daichi Shindo, Kazuki Fujiwara, Haruka Suzuki, Yuki Ueno, Hiroyuki Kato, Michihiro Takada, Yusuke Adachi, Manabu Todoroki, Susumu Iwasaki, Nobukazu Okimoto, Ken Nakata

**Affiliations:** 1 Department of Health and Sport Sciences, Graduate School of Medicine, Osaka University, Osaka 560-0043, Japan; 2 Department of Sports Medical Biomechanics, Graduate School of Medicine, Osaka University, Osaka 565-0871, Japan; 3 Institute of Food Sciences and Technologies, Ajinomoto Co., Inc., Tokyo 104-8315, Japan; 4 Research Institute for Bioscience Products & Fine Chemicals, Ajinomoto Co., Inc., Tokyo 104-8315, Japan; 5 Global Communications Department, Ajinomoto Co., Inc., Tokyo 104-8315, Japan; 6 Sports Nutrition Department, Ajinomoto Co., Inc., Tokyo 104-8315, Japan; 7 Sport and Health Science, Ritsumeikan University Graduate School, Kyoto 616-8001, Japan; gr0521rh@ed.ritsumei.ac.jp; 8 Department of Health and Human Performance, Fort Lewis College, Durango, CO 81301, USA; siwasaki@fortlewis.edu; 9 Okimoto Clinic, Hiroshima 734-0304, Japan

**Keywords:** visual analog scale, pain, joints, connective tissue

## Abstract

This short-term survey examined the effect of body part pain on subjective and objective handball performance in Japanese male national handball athletes. Fourteen athletes participated in this study. Assessments of pain in 10 body parts and subjective performance (*concentration* and *satisfaction with body movement*) were performed using a visual analog scale from 0 to 10 over four consecutive training days. Monitoring of heart rate and body acceleration during training was also performed to quantify the objective performance. Path analysis and linear mixed modeling were employed to assess the relationship between body pain scores and subjective/objective handball performance. Over the four days of the study period, the body part in which most athletes reported pain was the dominant shoulder (6 of 14 athletes), followed by the dominant knee, the dominant elbow, the dominant ankle joint, and the non-dominant ankle joint (3 of 14 athletes). The path analysis revealed that pain in the dominant elbow negatively correlated with concentration (standardized path coefficient = −0.644, *p* = 0.00), which was associated with satisfaction with body movement (standardized path coefficient = 0.704, *p* = 0.00). No significant effect of body pain on objective performance (heart rate and body acceleration) was found among the athletes in this study. The results suggested that the elite athletes were practicing with pain. Even if pain does not physically affect athletes’ objective performance, pain in the upper extremities, associated with the primary handball movement of throwing, may reduce the quality of practice by lowering athletes’ subjective performance.

## 1. Introduction

Handball, a globally recognized ball game, has achieved international acclaim and secured its status as an Olympic sport for both sexes [1]. This sport has permeated diverse regions, with national federations organized in Africa, Asia, Europe, America, the Caribbean, and Oceania [2]. Handball is played on a standardized 40-meter-by-20-meter court, and athletes demonstrate adept ball-handling skills as they strategically maneuver to throw shots into the opponent’s goal to accumulate points.

Handball is a physically demanding sport [3]. Intense physical contact is constantly repeated, especially around the goal area line. In addition, jump landings and change in directions are fundamental skills when performing jump shots and faking an action, which sometimes put a high-energy load on athletes’ lower limbs. Furthermore, athletes repeatedly throw a ball weighing 500 g at more than 100 km/h in several arm trajectories, placing a heavy burden on the shoulders and elbows. Since approximately 6 to 10 high-intensity shots are made per game [4], the accumulated minor stress on the upper limb joints might be significant [5,6]. Due to these game characteristics, handball is a sport in which acute trauma and chronic injury occur frequently [3,7], and these injuries sometimes seriously damage players’ careers. Therefore, it is essential to detect the signs of a potential injury and deal with any problems that arise before they become severe.

One possible sign of a subsequent problem is body part pain, such as shoulder pain and knee joint pain [8,9,10]. In particular, the accumulation of small pains is considered to be problematic because they tend to be underestimated [11,12] and may lead to chronic injury if proper treatments are not provided. However, for elite professional handball players, their situation sometimes does not allow them to be suspended from playing handball because, even if they have some pain, they may be able to compete without showing any negative effects on their physical athletic performance [13,14]. In addition, continuing to compete while in pain has become a way to maintain one’s identity as an elite athlete [13,14], and both the athletes themselves and the coaches who surround them have become desensitized to dealing with pain. Such situation may unwillingly overshadow the impact of pain on handball performance. Therefore, it is important to closely monitor how physical pain affects athletes’ behavior, including their subjective performance, such as their concentration level or satisfaction with body movement in their daily practice. To date, no study has evaluated how physical pain that does not lead to interruption of competition affects subjective and objective handball performance during actual national team practice. If it is confirmed that body part pain harms athletes’ subjective performance (e.g., concentration level or satisfaction with body movement) and results in physical performance loss, then body pain monitoring in daily practice will contribute to avoiding a reduction in their handball performance.

This study aimed to investigate how pain in various body parts affects subjective and objective handball performance during an actual training camp of a Japanese male national team. In general, a national team consists of a few selected elite players [15]. In addition, these athletes train at high intensity and prepare for international matches within a short preparation period, making it extremely difficult to establish a long and stable survey period. Recognizing such research limitations, this study aimed to identify the relationship between body part pain and subjective/objective handball performance over a short survey period through the use of a comprehensive visual analog scale (VAS) questionnaire at the end of each training session. The hypothesis was that body part pain would decrease athlete’s subjective concentration level during training and result in a lower satisfaction with body movement and physical performance loss.

## 2. Methods

### 2.1. Participant Information

Twenty-two male handball players were invited to a domestic training camp of the Japanese national handball team from 26 August to 4 September 2022 to prepare for an upcoming international match. The exclusion criteria were that players were not a member of the national team, and that players were unable to fully participate in training due to severe orthopedic trauma or poor health condition. Athletes who had no physical pain or, if they did, could still participate in all training sessions were candidates for inclusion. The research purpose and survey procedure were explained to all athletes, and 14 out of the 22 athletes agreed to participate in this study based on their own free will. During the training camp, these athletes’ usual means of pain control, i.e., supplements, medications, analgesic injections by a physician, and massage by a therapist, were not restricted and were recorded in detail. This study was approved by the Research Ethics Review Committee of Osaka University Hospital (No. 21457) and Ajinomoto Co., Inc. (No. 2021-025), and was registered at the University Hospital Medical Information Network Clinical Trials Registry under registration No. UMIN000048756. Written consent was obtained from all participants.

### 2.2. Data Collection

#### 2.2.1. Schedule and Survey Procedure

During the training camp held from 26 August to 4 September 2022, pain monitoring was performed during the second training session, i.e., 4 consecutive practice days from 29 August to 1 September 2022. The second session was chosen as the survey period because it was the longest session with consecutive practice days between rest days during this camp period, and the practice contents during these four days were constant and stable. The VAS questionnaire and other measurements detailed below were recorded by the experimenters (I.O., H.S., and Y.U.) during in-person interviews with the athletes to ensure accurate measurements.

#### 2.2.2. Athletes’ Basic Information

The athletes’ age (years), height (cm), weight (kg), handball experience (years), position (back/wing/pivot/goalkeeper), dominant hand (R/L), leg used for jumping (R/L), and regular use of any medications within one year were recorded at the beginning of the training camp.

#### 2.2.3. Quantification of Degree of Body Part Pain and Subjective Handball Performance

A total of 10 body parts, i.e., the dominant and non-dominant shoulders, elbows, knees, ankles, and Achilles tendons, were evaluated. Using a VAS, no pain was explained to the athletes as 0 and the greatest pain ever experienced was explained as 10. Further, the athletes were allowed to report the degree of pain in other body parts that they had. Two items of subjective handball performance (*Concentration* and *Satisfaction with body movement*) were also quantified using a VAS from 0 to 10. The VAS questionnaire was performed within 5 min after the closing of a practice of the day. The athletes answered the VAS questionnaire themselves with a pencil in the presence of the experimenters (I.O., H.S., and Y.U.).

#### 2.2.4. Measurement of Heart Rate and Body Movement Vigorousness

To evaluate objective physical performance, this study adopted the variables of *heart rate zone* and *body movement vigorousness*. To this end, this study measured the athletes’ heart rate, and acceleration due to body movement that was measured using a wearable heart rate inertial sensor (Polar Verity Sense, Polar Electro, Finland). The sensor was fixed to the non-dominant upper arm to avoid interfering with the players’ throwing motion. The heart rate data (sampling frequency: 1 Hz) and acceleration data (sampling frequency: 52 Hz, range ±8 G) obtained from the sensor were measured using the Polar Sensor Logger (https://play.google.com/store/apps/details?id=com.j_ware.polarsensorlogger (accessed on 18 February 2024)) and stored in an Android tablet (Galaxy Tab S7, Samsung Electronics Co., Ltd., Seoul, Republic of Korea) for subsequent data analysis.

### 2.3. Measurements

The handwritten VAS records of body part pain and subjective handball performance were quantified by two experimenters independently, and the mean value of the two experimenters’ measures was adopted as the final VAS score. The correctness of this value was confirmed by the third experimenter.

#### 2.3.1. Heart Rate Data

Heart rate data were measured only during a 15 min standardized warm-up period. The team’s warm-up practice consisted of the same exercises (e.g., light jogging, dynamic stretching, step work, and sprints) throughout the survey period and was instructed by the same athletic trainer. The main content of the practice varied slightly from day to day; so, the heart rate level was affected by both the practice intensity and the athletes’ physical condition. To evaluate the influence of the athletes’ physical condition, the heart rate level during standardized warm-up was used. Heart rate data were normalized to the maximum heart rate, and the staying time in each of the six heart rate bands (i.e., <50%, 50–59%, 60–69%, 70–79%, 80–89%, and ≥90%) was calculated as a percentage of the total warm-up time (15 min). For subsequent statistical analysis, this study used the heart rate bands of <50% and ≥90%. The maximum heart rate was calculated as 220 minus age [16].

#### 2.3.2. Body Acceleration Data

The body acceleration data obtained during the warm-up period (15 min) were analyzed. To evaluate the vigorousness of the athletes’ body movement, this study adopted the acceleration signal processing technique proposed by Marutani et al. (2023) [17]. This technique separately evaluates the high- and low-magnitude components of an acceleration signal by fitting a mixed Gaussian model onto the histogram of the appropriately processed acceleration signal. Briefly, the norm of the three-dimensional acceleration data obtained from the inertial sensor was calculated as $a=\sqrt{a_{x}^{2}+a_{y}^{2}+a_{z}^{2}}$, and the gravitational acceleration component was removed using a 2nd-order Butterworth digital filter (high-pass, zero time-shift, cutoff of 0.1 Hz). Then, the enveloped signal was obtained by using a second-order Butterworth digital filter (low-pass, cutoff of 1 Hz). The time periods when the envelope signal remained below 0.3 G for more than 5 s were regarded as the rest periods and excluded from further analysis. Afterwards, a histogram of the enveloped acceleration signal of the active phase was taken. Since this histogram of acceleration signal exhibited the characteristic of two local peaks that corresponded to low and high body movement vigorousness, this study applied mixed Gaussian model fitting onto the histogram. Body movement vigorousness was quantified with the acceleration values corresponding to the low- and high-magnitude peaks of the fitted mixed Gaussian model. Please see Marutani et al. (2023) [17] for a visual presentation of this signal processing method.

### 2.4. Statistical Analysis

This study aimed to evaluate the effects of body part pain on subjective and objective handball performance as accurately as possible, given the limitations of a small sample size and a short survey period during an actual training camp. To this end, a two-step statistical approach was used. First, this study used a path analysis to capture the overall relationship among the subjective and objective variables and examine the validity of our hypothesis. After excluding non-significant variables, this study then adopted a linear mixed model to account for the individual day-by-day variation in pain status and its effect on subjective performance during the survey days.

#### 2.4.1. Path Analysis of the Hypothesis Model

**Figure 1:**
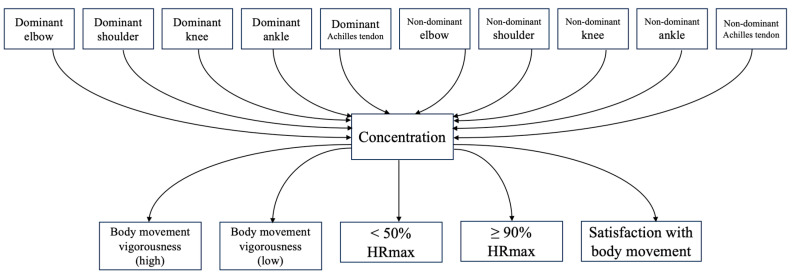
The initial hypothetical path model.

To obtain an overall causal relationship among the variables related to subjective handball performance (*concentration* and *satisfaction with body movement*) and objective physical performance (*heart rate zone* and *body movement vigorousness*), a path analysis was employed. The initial model based on our hypothesis is shown in Figure 1. The goodness of fit of the model was assessed by using a goodness-of-fit index (GFI) > 0.90, an adjusted goodness-of-fit index (AGFI) > 0.09, and a root-mean-square error of approximation (RMSEA) < 0.08 [18,19]. The Akaike information criterion (AIC) was used to assess the relative improvement of the goodness of fit once the model was updated. All statistical analyses in this phase were performed with Python (version 3.8.16). The library Semopy (version 2.3.9) was used for path analysis.

#### 2.4.2. Linear Mixed Modeling (LMM)

In addition to the path analysis, this study also applied linear mixed modeling to examine the effect of body part pain on concentration level while accounting for individual day-by-day variation. The following model was assessed using lmerTest (version 3.1.3) in R (version 4.2.3):

Concentration = dominant shoulder + non-dominant shoulder + dominant elbow + non-dominant elbow + dominant knee + non-dominant knee + dominant ankle + non-dominant knee + dominant Achilles tendon + non-dominant Achilles tendon + (1|Athlete),

where (1|Athlete) specified the grouping of each athlete.

## 3. Results

The demographic information of the participants is detailed in Table 1.

### 3.1. VAS Scores for Body Part Pain

The VAS scores for pain in different body parts are detailed in Table 2, with the color grading reflecting the magnitude of the VAS scores. Over the four days of the study period, the body part that most athletes reported pain was the dominant shoulder (6 of 14 athletes), followed by the dominant knee, the dominant elbow, the dominant ankle joint, and the non-dominant ankle joint (3 of 14 athletes). These body parts were the only ones that were consistently reported as painful throughout the survey period. Two athletes reported pain in the non-dominant shoulder, one in the non-dominant Achilles tendon, and one in the non-dominant knee. Still, pain in these body parts was temporary rather than persistent throughout the entire survey period. Particularly, the dominant knee of athlete No. 14 and the non-dominant elbow of athlete No. 10 recorded high pain VAS scores throughout the survey period. As no athlete reported pain in the dominant Achilles tendon, the VAS score of this body part was excluded from further analysis.

### 3.2. Path Analysis Using Subjective and Objective VAS Scores

**Figure 2:**
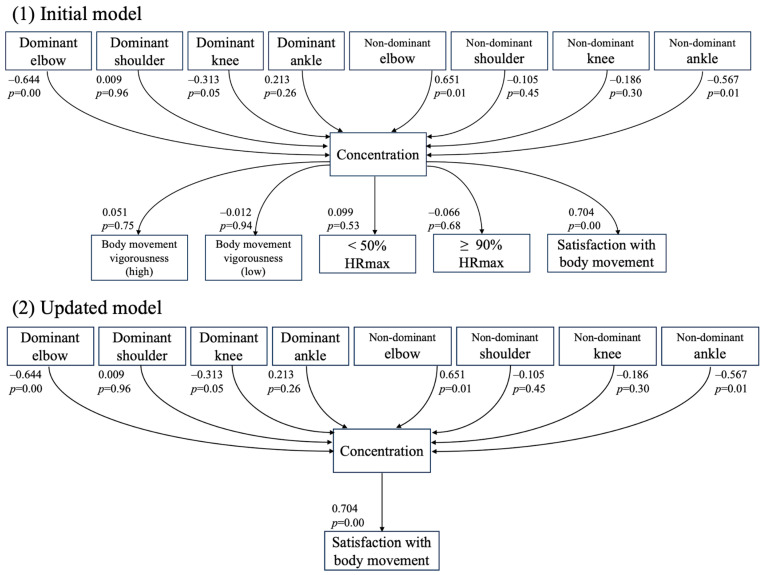
The results of the path analysis.

The VAS scores for subjective (*concentration* and *satisfaction with body movement*) and objective performance (*heart rate zones* and *body movement vigorousness*) are shown in Table 3 and Table 4, respectively. An initial path analysis model predicting subjective and objective VAS scores from pain VAS scores was established (Figure 2(1)). The initial model (GFI = 0.75, AGFI = 0.70, RMSEA = 0.02, and AIC = 45.9) showed that pain in the dominant elbow (−0.644, *p* = 0.00) and non-dominant ankle (−0.567, *p* = 0.01) negatively affected *concentration*. However, pain in the non-dominant elbow (0.651, *p* = 0.01) positively affected *concentration*. Although *concentration* positively affected *satisfaction with body movement* (0.704, *p* = 0.00), it did not show any significant correlations with objective *heart rate zones* (<50%: 0.099, *p* = 0.53, and >90%: −0.066, *p* = 0.68) and *body movement vigorousness* (high: 0.051, *p* = 0.75, and low: −0.012, *p* = 0.94). The inclusion of objective performance (*heart rate zones* and *body movement vigorousness*) reduced the goodness of fit of the model. The updated final model, which excluded *heart rate zones* and *body movement vigorousness*, showed increased goodness-of-fit scores (GFI = 0.92, AGFI = 0.90, RMSEA = 0, and AIC = 29.4; Figure 2(2)).

### 3.3. Linear Mixed Modeling (LMM)

Based on the result of the final path model, this study additionally employed LMM to account for individual day-by-day variation in pain status. The LMM indicated that pain in the dominant elbow negatively affected concentration (−0.36, *p* = 0.02, Table 5). Overall, pain in the dominant elbow was indicated by both the path analysis and LMM as a contributor to subjective performance loss.

## 4. Discussion

### 4.1. Aim and Hypothesis

This study aimed to investigate how body part pain affected subjective handball performance (*concentration* and *satisfaction with body movement*) and objective performance (*heart rate and body movement vigorousness*) during a single training session at a training camp for a male Japanese national team. In such a national team, which is organized for a short period and trains at high intensity, it is difficult to conduct a long-term survey. Therefore, short-term monitoring is required to determine players’ physical condition as accurately as possible. It was hypothesized that body part pain would interfere with players’ subjective and objective handball performance during the training camp.

### 4.2. Summary of Pain Results

Pain was recorded at different degrees of severity depending on the body parts. Three players reported a severe pain score of 7.0 or higher in their dominant knee on the VAS throughout the survey period (Table 2). The ankle joints were also painful for four players, one of whom (No. 08) had pain in both ankle joints. The VAS scores for ankle joint pain were also relatively high, with a maximum score of 8.5. The ankles and knee joints are the body parts that most frequently experience acute orthopedic trauma in handball [6,9,10,20], and insufficient recovery typically results in overuse symptoms [6]. On the other hand, eight athletes reported pain in their upper-extremity joints, including the dominant elbow and shoulder. Although the maximum VAS score of about 7.0 for pain in the upper-extremity joints was not as high as that for pain in the lower-extremity joints, it was evident that these athletes were concerned about chronic upper-extremity pain throughout the survey period. Eight players had no apparent history of acute trauma to their dominant elbow or shoulder. It was speculated that the repetitive high-intensity throwing motion in handball was the trigger for such chronic upper-extremity pain.

### 4.3. Pain in Dominant Elbow Reduced Subjective Performance

Two statistical models (the path model and the LMM) supported that pain in the dominant elbow reduced the athletes’ concentration during training. A possible limitation of the path model was that individual day-by-day variation in the repeated measurements was not reflected in the results. In contrast, the LMM took individual day-by-day variation into account and obtained consistent results with the path analysis, thus confirming the negative effect of pain in the dominant elbow on subjective handball performance. Of the 14 players, 3 players reported pain in the dominant elbow, all related to throwing. Among the upper extremities, the number of pain issues complained for the dominant shoulder was higher. Still, the VAS scores for pain in the dominant elbow were slightly higher (Table 2). This might contribute to a lower satisfaction with throwing-related play, leading to lower concentration. Specifically, elbow pain may contribute to athletes’ frustration that comes from a series of unwilling decisions due to their hesitation to make solid shots and long passes. Although pain in the goalkeepers’ elbow is a typical elbow problem in handball [21,22,23], the overhead-throwing-related elbow problem of court players is also an issue in handball [24,25]. Court players must configure a better throwing arm trajectory in response to interference from the opposing defenders or goalkeepers. Such handball-specific requirements in throwing do not always allow athletes to perform anatomically with safe manner and comfortable throwing kinematics. Pain in the dominant elbow should be monitored as a risk sign that may lead to poor athletes’ satisfaction.

### 4.4. Lower Limb Joint Pain Did Not Affect Subjective Performance

It was somewhat surprising that pain in the knees and ankles was not shown to have a significant relationship with subjective handball performance in the path analysis (Figure 2), although the knees and ankles were recorded to have high pain VAS scores (Table 2). This is not necessarily a reflection of a universal fact for all handball players as it is a result obtained from a small number of national team athletes complaining of knee and ankle pain during the survey period. Since handball is a sport that involves rapid change of direction movements and repeated jumps and landings, it is easy to imagine that stress on the muscle–tendon units around the lower limb joints is high. To clarify the relationship between pain in the lower-extremity joints and subjective handball performance, studying a larger number of athletes for a more extended period to observe day-by-day changes in both pain status and subjective handball performance would be necessary.

### 4.5. Body Movement Vigorousness Was Not Affected by Body Pain

The hypothesis that body movement vigorousness, as quantified by the inertial sensor, would show changes linked to body part pain was not supported by the results of the path analysis. This might be because the pain experienced by the national team athletes in this training camp was not severe enough to reduce their physical performance. The ability to exhibit stable physical performance in the presence of pain is considered an ability of national team athletes. Still, it carries the risk of missing signs of pain. In the stage when pain progresses and manifests itself as a chronic disorder, the vigorousness of body movement is expected to be noticeably reduced. This may also be due to the responsibility of national team athletes to prepare for international competitions during their short training camps. It is thought that experienced athletes, such as those in a national team, cannot show a loss in physical performance if they are in a bit of pain, although such pain tolerance may trigger chronic disability in the long term. As a clinical implication, it is essential to keep an eye on the variability in athletes’ status changes in pain, subjective handball performance, and physical performance during daily monitoring so as to not underestimate the consequence of pain of a degree that is not enough to reduce physical performance.

### 4.6. Limitations

This study had several limitations. Although this study aimed to observe natural athletes’ behavior during a training camp, the small sample size and biased sampling of elite athletes might make it difficult to generalize the findings to general handball athletes. However, what this study can suggest to handball teams in general is that a careful quantification of body pain and subjective handball performance might help explain the impact of body pain on concentration and satisfaction with handball performance, and this might help identify the risk sign of a potential chronic injury before it becomes significant. The lack of a control group was also a limitation of this survey study with elite athletes. It was difficult to recruit physically matched pain-free athletes for such a comparison. Future studies may want to recruit athletes with a wide range of performance level to solve such limitations. Because this study was a short-term survey due to the constraints of the camp schedule, it was not possible to monitor pain variability over a long period of time. A possible solution to monitor body part pain independently from a camp’s schedule is to adopt the Web-survey approach [26]. Despite these research limitations, this study used the VAS assessment in face-to-face interviews with athletes to accurately quantify subjective sensation of pain. In addition, four days of constant practice at high intensity were selected as the study period to eliminate the influence of practice intensity on pain variability as much as possible. Therefore, it is considered that a short duration of the survey period and a small number of athletes recruited are not reasons to hesitate to conduct careful evaluations of athletes’ conditions.

## 5. Conclusions

This open-field study investigated the effect of body part pain on subjective (*concentration* and *satisfaction with body movement*) and objective (*heart rate* and *body acceleration*) performance during the training camp of a male Japanese national team. Over the four days of the study period, the body part in which most athletes reported pain was the dominant shoulder (6 of 14 athletes), followed by the dominant knee, the dominant elbow, the dominant ankle joint, and the non-dominant ankle joint (3 of 14 athletes). The path analysis and LMM revealed that pain in the dominant elbow negatively correlated with concentration (standardized path coefficient = −0.644, *p* = 0.00), which was associated with satisfaction with body movement (standardized path coefficient = 0.704, *p* = 0.00). No significant effect of body pain on objective performance was found. The elite athletes in this study were observed to practice with pain. Even if pain does not physically affect athletes’ objective performance, pain in the upper extremities, associated with the primary handball movement of throwing, may reduce the quality of practice by lowering athletes’ subjective performance.

## Figures and Tables

**Table 1:** Demographic information of the participants.

No.	Age (y.o.)	Height (cm)	Weight (kg)	Position	Experience (Years)	Dominant Arm/Leg
1	26	190	83.9 [0.4]	GK	13	R/R
2	22	190	89.8 [0.6]	RW, RB	14	L/R
3	22	182	82.6 [0.2]	RW	15	L/R
4	28	178	80.1 [0.5]	LW	18	R/L
5	22	186	93.6 [0.6]	LB, CB	8	R/L
6	32	183	97.4 [0.4]	RB, RW	20	L/R
7	27	190	85.6 [0.4]	GK	12	R/L
8	26	183	87.3 [0.6]	LB	18	R/L
9	22	190	84.2 [0.4]	LB	11	R/L
10	25	190	99.8 [0.6]	PV	12	R/L
11	22	186	91.6 [0.5]	RB, CB, LB	9	R/L
12	29	182	83.4 [0.3]	CB	22	R/L
13	31	182	86.1 [0.7]	GK	20	R/L
14	33	190	97.7 [0.0]	LB	20	R/L

Weight is expressed as the mean [standard deviation] of 4 survey days, measured after waking up. GK: goalkeeper; LW: left wing; RW: right wing; LB: left back; CB: center back; RB: right back; and PV: pivot. Dominant Arm: the main hand used to make a shot. Dominant leg: the main leg used in a jump shot.

**Table 2:** VAS scores for body part pain of all athletes during the entire training camp period.

	Dominant Knee				Dominant Achilles tendon				Dominant Ankle				Dominant Elbow				Dominant Shoulder						
No.	D1	D2	D3	D4	D1	D2	D3	D4	D1	D2	D3	D4	D1	D2	D3	D4	D1	D2	D3	D4			
1	0	0	0	0	0	0	0	0	0	0	0	0	0	0	0	0	0	0	0	0			
2	0	0	0	0	0	0	0	0	0	0	0	0	0	0	0	0	0	0	0	0			
3	0	0	0	0	0	0	0	0	0	0	0	0	0	0	0	0	0	0	4.3	0			
4	7.2	8	2.5	4.5	0	0	0	0	0	0	0	0	0	0	0	0	0	0	0	0			
5	2.7	3.6	2.8	1.8	0	0	0	0	6.4	3.6	3	1.8	0	0	0	0	2.9	4	0	1.5			
6	0	0	0	0	0	0	0	0	0	0	0	0	5.9	6.6	6	7.1	0	0	0	0			
7	0	0	0	0	0	0	0	0	0	0	0	0	0	0	0	0	0	0	0	0			
8	0	0	0	0	0	0	0	0	5.7	8.5	5.8	5.9	0	0	0	0	5	6.2	4.1	4.2	Pain VAS score		
9	0	0	0	0	0	0	0	0	0	0	0	0	0	6.2	0	0	3.1	3.7	0	0	range in color		
10	0	0	0	0	0	0	0	0	0	0	0	0	0	5.7	6.5	6.2	0	0	0	0		[8, 10]	
11	0	0	0	0	0	0	0	0	0	0	0	0	0	0	0	0	5	3.5	4.8	2.8		[6, 8)	
12	8.3	9.1	10	7.8	0	0	0	0	0	0	0	0	0	0	0	0	0	0	0	0		[4, 6)	
13	0	0	0	0	0	0	0	0	0	0	0	0	0	0	0	0	1.9	2.8	3.2	1.8		[2, 4)	
14	-	0	0	0	-	0	0	0	-	3	0	0	-	0	0	0	-	0	0	0		(0, 2)	
	Non-dominant Knee				Non-dominant Achilles tendon				Non-dominant Ankle				Non-dominant Elbow				Non-dominant Shoulder						
No.	D1	D2	D3	D4	D1	D2	D3	D4	D1	D2	D3	D4	D1	D2	D3	D4	D1	D2	D3	D4			
1	0	0	0	0	0	0	0	0	0	0	0	0	0	0	0	0	0	0	0	0			
2	0	0	0	0	0	0	0	0	0	0	0	0	0	0	0	0	0	0	0	0			
3	0	0	0	0	0	0	0	0	0	0	0	0	0	0	0	0	0	0	0	0			
4	0	0	0	0	0	0	0	0	0	0	0	0	0	0	0	0	0	0	0	0			
5	0	0	0	0	0	0	0	0	0	0	0	0	0	0	0	0	0	0	0	1.6			
6	0	0	0	0	0	0	0	0	0	0	0	0	0	0	0	0	0	0	0	0			
7	0	0	0	0	0	0	0	0	3.9	7.2	4.7	5.3	0	0	0	0	0	0	0	0			
8	0	0	0	0	0	0	0	0	0	0	0	0	0	0	0	0	0	0	0	0			
9	0	0	0	0	0	0	0	0	0	0	5.7	3.7	0	0	0	0	0	0	0	0			
10	0	0	6.4	0	0	0	0	0	6.7	5.8	0	6.6	6.3	5.7	6.5	6.2	0	0	0	0			
11	0	0	0	0	0	0	0	0	0	0	0	0	0	0	0	0	0	0	0	0			
12	0	0	0	0	1.6	1.1	0	4.6	0	0	0	0	0	0	0	0	0	0	0	0			
13	0	0	0	0	0	0	0	0	0	0	0	0	0	0	0	0	0	0	0	0			
14	-	0	0	0	-	0	0	0	-	0	0	0	-	0	0	0	-	0	2.7	2.9			

Note: athlete No. 14 joined the training camp on day 2. ( ) in the pain VAS score range indicates “more than” and “less than”. [ ] indicates “or more” and “or less”.

**Table 3:** VAS scores for concentration and satisfaction with body movement.

	Concentration				Satisfaction with Body Movement			
No.	Day 1	Day 2	Day 3	Day 4	Day 1	Day 2	Day 3	Day 4
1	3.5	7.5	3.5	3.8	2.9	7.5	3.5	3.9
2	7.4	3.5	3.9	3.5	7.5	2.8	3.1	2.2
3	6.7	7.3	5.7	6.7	7.3	7.3	6.4	6.8
4	7.6	7.7	7.7	6.9	7.6	7.8	7.8	6.7
5	7.5	6.0	7.5	7.9	5.6	5.3	4.9	5.2
6	4.2	6.7	4.3	2.6	5.6	6.5	5.4	4.1
7	3.8	3.5	2.5	2.6	4.0	3.8	2.6	2.5
8	5.5	6.0	6.1	5.6	5.5	5.3	5.7	4.8
9	6.1	2.8	5.5	4.6	6.1	3.5	3.9	5.6
10	6.8	6.4	6.1	5.7	6.9	6.3	6.1	5.5
11	7.6	7.5	10.0	8.0	5.6	5.2	10.0	7.8
12	3.3	7.4	2.0	5.4	5.0	5.4	2.9	2.6
13	4.3	6.5	7.5	7.5	2.2	5.6	6.3	6.3
14	-	0.0	5.0	6.8	-	2.9	2.0	5.3

Note: athlete No. 14 joined the training camp on day 2.

**Table 4:** Objective handball performance variables evaluated with a wearable sensor.

	Body Movement Vigorousness (High) Unit: G				Body Movement Vigorousness (Low) Unit: G				<50% HRmax Unit: %				>90% HRmax Unit: %			
No.	Day 1	Day 2	Day 3	Day 4	Day 1	Day 2	Day 3	Day 4	Day 1	Day 2	Day 3	Day 4	Day 1	Day 2	Day 3	Day 4
1	1.1	1.1	1.2	1.0	0.3	0.3	0.4	0.3	5.4	1.1	11.9	17.4	0.5	5.6	1.6	0.0
2	1.2	1.2	1.1	1.1	0.4	0.3	0.4	0.3	0.0	0.3	11.8	9.1	14.4	4.3	0.1	0.0
3	1.3	1.3	1.2	1.2	0.4	0.4	0.3	0.3	10.1	3.4	28.9	23.3	0.0	0.0	0.0	0.0
4	1.3	1.1	1.4	1.3	0.4	0.4	0.4	0.3	3.4	0.6	1.4	11.5	32.4	26.9	12.0	0.6
5	1.2	N.A.	1.1	1.0	0.3	N.A.	0.3	0.3	6.3	N.A.	3.3	12.3	24.0	N.A.	23.3	0.0
6	1.3	1.1	1.2	1.2	0.3	0.3	0.3	0.3	7.4	3.0	33.3	12.8	6.6	4.5	0.1	0.0
7	N.A.	N.A.	1.0	1.1	N.A.	N.A.	0.3	0.3	N.A.	N.A.	7.5	9.7	N.A.	N.A.	4.5	9.4
8	1.1	1.1	1.2	N.A.	0.3	0.3	0.3	N.A.	5.8	1.3	10.4	N.A.	5.0	12.4	13.0	N.A.
9	1.1	1.2	1.3	1.1	0.3	0.3	0.3	0.3	5.9	0.9	3.8	11.3	1.1	1.6	4.2	0.0
10	1.3	1.1	1.1	1.2	0.3	0.3	0.3	0.3	5.3	3.0	6.8	14.3	4.2	3.0	1.1	0.0
11	1.2	1.1	1.2	1.2	0.3	0.3	0.3	0.3	13.6	20.8	15.4	34.4	0.5	0.7	0.0	0.0
12	1.2	1.1	1.3	1.1	0.3	0.3	0.3	0.3	7.2	1.3	3.6	7.1	21.1	23.3	16.7	0.9
13	1.4	1.2	1.3	1.3	0.4	0.3	0.3	0.3	2.4	0.0	10.8	9.1	8.7	8.3	2.3	6.5
14	-	N.A.	1.2	1.2	-	N.A.	0.3	0.3	-	N.A.	5.1	15.9	-	N.A.	1.7	0.0

Note: some athletes’ wearable sensors lost connection with the Android tablet during the warm-up phase. Thus, their performance scores, which are represented as N.A., were not calculated. Those athletes were excluded from the path analysis and mixed linear modeling. Athlete No. 14 joined the training camp on day 2.

**Table 5:** Results of the linear mixed model.

	Estimate	Standard Error	Degree of Freedom	t-Value	*p*-Value
Intercept	6.757	0.640	12.207	10.547	<0.01 *
D Shoulder	−0.042	0.179	28.854	−0.236	0.82
Nd Shoulder	0.108	0.435	24.926	0.248	0.80
D Elbow	−0.358	0.142	22.842	−2.520	0.02 *
Nd Elbow	0.324	0.323	10.830	1.003	0.34
D Knee	−0.156	0.134	13.824	−1.166	0.26
Nd Knee	−0.076	0.323	32.983	−0.236	0.81
D Ankle	0.044	0.212	12.185	0.207	0.84
Nd Ankle	−0.169	0.209	23.113	−0.810	0.43
Nd Achilles Tendon	0.183	0.348	31.939	0.528	0.60

D: dominant, Nd: non-dominant. Asterisk (*) indicates statistical significance (*p* < 0.05).

## Data Availability

Data that support the findings of this study will be made available from the corresponding author upon reasonable request. The data are not publicly available due to the participants’ wishes.
